# Is Malignant Potential of Barrett’s Esophagus Predictable by Endoscopy Findings?

**DOI:** 10.3390/life10100244

**Published:** 2020-10-16

**Authors:** Yuji Amano, Norihisa Ishimura, Shunji Ishihara

**Affiliations:** 1 Department of Endoscopy, New Tokyo Hospital, 1271 Wanagaya, Matsudo, Chiba 270-2232, Japan; 2 Department of Internal Medicine II, Faculty of Medicine, Shimane University, Shimane 693-8501, Japan; ishimura@med.shimane-u.ac.jp (N.I.); si360405@med.shimane-u.ac.jp (S.I.)

**Keywords:** Barrett’s esophagus, esophageal adenocarcinoma, endoscopic predictor, biomarkers, endoscopic surveillance

## Abstract

Given that endoscopic findings can be used to predict the potential of neoplastic progression in Barrett’s esophagus (BE) cases, the detection rate of dysplastic Barrett’s lesions may become higher even in laborious endoscopic surveillance because a special attention is consequently paid. However, endoscopic findings for effective detection of the risk of neoplastic progression to esophageal adenocarcinoma (EAC) have not been confirmed, though some typical appearances are suggestive. In the present review, endoscopic findings that can be used predict malignant potential to EAC in BE cases are discussed. Conventional results obtained with white light endoscopy, such as length of BE, presence of esophagitis, ulceration, hiatal hernia, and nodularity, are used as indicators of a higher risk of neoplastic progression. However, there are controversies in some of those findings. Absence of palisade vessels may be also a new candidate predictor, as that reveals degree of intense inflammation and of cyclooxygenase-2 protein expression with accelerated cellular proliferation. Furthermore, an open type of mucosal pattern and enriched stromal blood vessels, which can be observed by image-enhanced endoscopy, including narrow band imaging, have been confirmed as factors useful for prediction of neoplastic progression of BE because they indicate more frequent cyclooxygenase-2 protein expression along with accelerated cellular proliferation. Should the malignant potential of BE be shown predictable by these endoscopic findings, that would simplify methods used for an effective surveillance, because patients requiring careful monitoring would be more easily identified. Development in the near future of a comprehensive scoring system for BE based on clinical factors, biomarkers and endoscopic predictors is required.

## 1. Introduction

In Western populations, patients with esophageal adenocarcinoma (EAC) derived from Barrett’s esophagus (BE) have shown a marked increase in recent decades. Furthermore, the clinical outcome of EAC has been reported that the prognosis worsens as the stage progresses, although a 5-year survival is around 47% for localized EAC [1,2,3,4]. The annual incidence of EAC arising from BE has reported to be 0.33% in a meta-analysis [5], and 0.36% in a prospective study [6]. Desai M at al. reported there was a significant increase in the detection of high-grade dysplasia and EAC (1990–1994, 5.1%; to 2005–2009, 6.3%; and 2010+, 16.3%) [7]. Thus, calculated rates of incidence of neoplastic progression in BE cases have never shown a reduction, this been suggested that endoscopic surveillance is essentially required for affected patients, as stated in guidelines presented by the British Society of Gastroenterology and American Colleague of Gastroenterology [8,9]. The American Society for Gastrointestinal Endoscopy (ASGE) standard of practice committee has reported that most patients with EAC are diagnosed in a later stage of the disease and performance of BE surveillance is associated with a 25% reduction in mortality [10]. However, endoscopic surveillance of BE is considered to be laborious and costly, as the protocol requires obtaining several biopsy specimens from quadrant areas, each with a 1 or 2 cm interval. Therefore, a more effective and simpler endoscopic surveillance method for clinical practice is anticipated to assist management of patients with BE.

Understanding of available information regarding the potential of neoplastic progression in BE prior to endoscopic surveillance is inevitably important. While several clinical risk factors, such as male gender, aging, smoking, high-fat diet, obesity, reflux esophagitis, colon neoplasms, as well as others, are well-known important predictors of Barrett’s carcinogenesis [11,12,13,14,15,16,17,18], they are not considered to be fully reliable indicators of neoplastic progression in all patients with BE. However, presence of erosive esophagitis or recurrent gastroesophageal reflux disease (GERD) symptom have been reported to be important predictors for neoplastic progression in BE as described below [19,20], and subsequently, proton pump inhibitor (PPI) use was protective against the progression [12,21,22]. Recently, biomarkers including epigenetics and miRNA analysis findings, DNA content abnormalities, and loss of heterozygosity noted in biopsy findings have been reported to be additional reliable predictors of malignant transformation in affected patients [23]. One of the most important biomarkers is aberrant expression and/or mutation of p53 [24,25]. Some of these biomarkers become to be detectable by specialized endoscopic devices such as autofluorescence endoscopy, optical coherence tomography, endocytoscopy, confocal endomicroscopy, or near-infrared imaging endoscopy, although use of these novel markers is difficult in clinical settings because of the complicated biochemical procedures required. On the other hand, various findings obtained with white light endoscopy (WLE), such as length of BE segment and presence of esophagitis, ulceration, and hiatal hernia, along with others, can be used for prediction of progression to EAC. Nevertheless, such endoscopic findings must be verified for use in clinical practice before being considered available as a true marker for neoplastic progression. Another significant factor related to Barrett’s carcinogenesis is cyclooxynase-2 (COX-2) protein expression, which has been reported to be a key event in transformation to a dysplastic lesion [26]. Endoscopic information regarding COX-2 protein expression in BE case can be detected by some endoscopic methods including chromoendoscopy or narrow band imaging (NBI) endoscopy, thus endoscopic surveillance may become to be a more efficacious by the use of these developed devices.

With the development of endoscopic methods and devices, the easier detection of neoplastic lesions of BE may be currently applicable for the clinical practice. Among them, NBI endoscopy that can be evaluated micro-mucosal and micro-vascular patterns is considered to be one of the representative candidates. In the present review, the contributions of endoscopic findings including WLE and image-enhanced endoscopy (IEE): chromoendoscopy, acetate-enhanced endoscopy, NBI endoscopy, and molecular imaging endoscopy, for prediction of the malignant potential of BE, were investigated. Furthermore, a new concept for efficient endoscopic surveillance of BE along with a targeting biopsy method instead of a random biopsy procedure is also discussed. Findings presented in this review were obtained with procedures conducted in accordance with the Declaration of Helsinki.

## 2. Possibility of WLE Findings for Predicting Neoplastic Progression

### 2.1. Length of BE

In the present review, according to the Prague C & M criteria [27], BE less than 3 cm in length was defined as short segment BE (SSBE), and more than 3 cm was done as long segment BE (LSBE). WLE findings that may indicate the risk of progression to EAC are shown in Table 1. It appears that a larger area of BE provokes a higher rate of EAC incidence. Although Rudolph RE et al. reported that the risk for EAC in patients with SSBE was not substantially lower than that in those with longer segments in a previous study [28] and Cameron AJ et al. did that BE carcinogenesis derived from presence of SIM irrespective of BE length by small EAC analysis [29], several recent studies demonstrated that EAC risk per BE length was higher in LSBE than SSBE as shown in Table 1. For instance, several studies have revealed that the risk of neoplastic progression increases by 1.11- to 1.39-fold with every 1 cm of length [30,31,32,33,34]. Holmberg D et al. also showed that an increase in maximum BE length was associated with increased risk of EAC (odds ratio (OR) 2.3, 95% confidence interval (CI) 1.4–3.9 for segments 3–8 cm in length; OR 4.3, 95% CI 2.5–7.2 for segments ≥ 8 cm) [35]. Thus, LSBE has revealed a higher incidence of EAC progression compared to SSBE as other investigators reported [32,36,37]. However, in general, progression to a dysplastic lesion occurs not only in patients with LSBE but also in those with SSBE, as another population-based study found no relationship between BE segment length and risk of dysplastic progression (hazard ratio (HR) 2.31, 95% CI 0.89–6.01) [38].

### 2.2. Other Endoscopic Findings

Erosive esophagitis, mucosal nodularity, Barrett’s ulcer and stricture are well-known representative factors related to risk of progression to EAC. Coleman HG et al. reported that presence of ulceration in Barrett’s segment was associated with EAC progression, with a HR of 1.72 (95% CI 1.08–2.76) [36]. Rugge M et al. also noted that patients with Barrett’s ulcer have a significant risk of neoplastic progression (relative risk [RR] 7.60, 95% CI 2.63–21.9) [31], and Sikkema M et al. found that the risk of BE carcinogenesis was increased by 3.5 fold (RR 3.5, 95% CI 1.3–9.5) when erosive esophagitis was present [20]. In contrast, Coleman HG et al. reported that no evidence of a relationship between erosive esophagitis and EAC progression was found in their study [36]. An endoscopic mucosal nodularity defined by Buttar NS et al. was a subtle mucosal elevation of 1 cm or less in diameter [39] and Solanky D et al. also concluded that the presence of mucosal nodularity is a significant predictor for EAC progression (HR 4.98, 95% CI 1.80–11.7) [34]. However, endoscopic findings indicating the nodularity should be given close attention for the diagnosis, since it may reflect prevalent neoplastic lesions of BE rather than a marker of future progression [40]. Hillman LC et al. reported that patients with one of severe esophagitis, nodularity, ulcer, or stricture as a marker had a 6.7 times (HR 6.7; 95% CI 1.3–35) greater risk, while those with two or more of those markers had a 14.1 times (HR 14.1; 95% CI 2.02–102) greater risk for development of EAC [41].

Hiatal hernia is also a well-documented risk factor for development of not only BE but also EAC, as Weston AP et al. suggested that a larger sized hiatal hernia was associated with increased risk of dysplastic changes in BE cases [30]. In contrast, no relationship between hiatal hernia and neoplastic progression was found in other related studies [20,32,42]. Therefore, even though BE segment length and Barrett’s ulcer have been proven to be undoubted predictors for neoplastic progression of BE, other candidate endoscopic findings remain controversial regarding their suitability as predictors of neoplastic progression in affected patients. Thus, LSBE has been confirmed to possess a high malignant potential and therefore surveillance is indispensable, although it is still unclear what kinds of SSBE reveal a high risk of neoplastic progression. In Asian patients, most of EAC are arising from SSBE, therefore to know a malignant potential of SSBE is also very interest of.

## 3. Endoscopic Detection of Intestinal Metaplasia

### 3.1. Detection by IEE

IEE in addition to WLE believes to make other important findings clear. One of the representative advantages of IEE is to prove the presence of specialized intestinal metaplasia (SIM). SIM is defined as an intestinal metaplasia with goblet cells in esophageal squamous epithelium, and it is an essential requirement for the histological confirmation of BE in some Western countries, such as the United States and Germany, though not England and Japan, and it is also known to be an important predictor of malignant potential in Barrett’s carcinogenesis cases [29,38,43,44]. Detection of SIM using IEE method, which includes chromoendoscopy, acetate-enhanced endoscopy, and NBI endoscopy, has recently been clinically investigated. Cant MI et al. reported that EAC was more significantly diagnosed by the biopsy specimens from methylene blue (MB) stained area than un-stained one (12% vs. 6%, *p* = 0.004) [45]. Thus, MB chromoendoscopy appears useful, since MB-targeted biopsy method is more accurate and cost-effective than random biopsy one. Additionally, Endo T et al. showed that MB chromoendoscopy with magnified observation was able to detect SIM with high sensitivity and make the mucosal pit pattern visible for classification, classified into the following five patterns: straight, dot, oval, tubular and villous [46]. Tubular and villous patterns frequently indicate the presence of SIM. However, it was demonstrated that MB chromoendoscopy was not superior for endoscopic surveillance in clinical practice because of non-specific MB staining for SIM [47,48]. On the other hand, Guelrud M. et al. demonstrated that four mucosal pit patterns: round, reticular, villous and ridged, could be found by acetate enhancement endoscopy, and SIM was frequently detectable by biopsy sample obtained from area with villous or ridged mucosal patterns [49]. Thus, acetate-enhanced magnifying endoscopy may be also applicable for efficient surveillance of BE via novel detection of SIM.

### 3.2. Detection by NBI Endoscopy

Hamamoto Y. et al. were the first to report that NBI endoscopy can provide information regarding micro-surface and micro-vascular patterns of BE and EAC [50]. They evaluated the quality of images for the visualization of the esophagogastric junction, capillary vessels, and columnar-lined esophagus (CLE) using scoring system by NBI endoscopy and WLE, and concluded that the visualization of the CLE was better by NBI endoscopy than by WLE, since net-like blood vessels were more clearly seen on images obtained by NBI endoscopy. Thereafter, Goda K. et al. proposed a classification by use of magnifying NBI endoscopy findings that made it possible to selectively diagnose SIM and Barrett’s dysplastic lesion with high sensitivity and specificity [51], which was later followed by other classifications [52,53,54,55]. These classifications are undoubtedly superior to detect SIM or dysplastic Barrett’s lesions with high sensitivity and specificity. However, they have not shown an appropriate level of diagnostic concordance, not only with trainees but also expert endoscopists, therefore they are needed to determine whether they can be widely accepted endoscopists with varying levels of expertise [56].

Nevertheless, it is important to note that Japanese and German pathologists have reported Barrett’s carcinogenesis that developed in background mucosa adjacent to EAC in many patients was cardiac type (approximately 60–70%) rather than intestinal type [57,58], which is one of the reasons why some patients with SSBE also possess a high cancer risk [59,60]. In these studies, Barrett’s mucosa was histologically confirmed by presence of esophageal glands proper and/or ducts, squamous islands, double muscularis mucosae, and palisade vessels [61], and Barrett’s mucosa adjacent to EAC was further divided into intestinal and cardiac type according to presence or absence of SIM detected by Alcian blue histochemical staining. Although SIM can be accurately detected by use of some IEE techniques, such detection may not be directly associated with prediction of malignant potential in BE cases.

## 4. Endoscopic Findings Related to COX-2 Protein Expression and Cellular Proliferation

It is well known that COX-2 protein expression plays an important role in Barrett’s carcinogenesis [26]. COX-2 protein is expressed by acid and/or bile exposure in Barrett’s mucosa, and subsequently activates cytokines, interleukins, growth factors, and tumor promoters [62]. Prostaglandin E2 is also induced by COX-2 protein expression and then stimulates cell proliferation and angiogenesis, and inhibits apoptosis, and these phenomena been strongly associated with carcinogenesis [63,64,65]. Therefore, chemoprevention of EAC by use of aspirin or non-steroidal anti-inflammatory drugs as well as selective COX-2 inhibitors has been a recent topic of interest [66,67]. In this carcinogenic pathway, expressions of COX-2 protein and prostaglandin E2, cellular proliferation, apoptosis, and angiogenesis are considered to be factors applicable as significant markers of malignant potential in cases of BE. Recently, some endoscopic findings including esophageal palisade vessels (PVs), mucosal pattern and micro-vascular pattern have been reported to possibly possess the correlation with COX-2 protein expression in BE as described below.

### 4.1. Assessment by Esophageal Palisade Vessels

**Figure 1:**
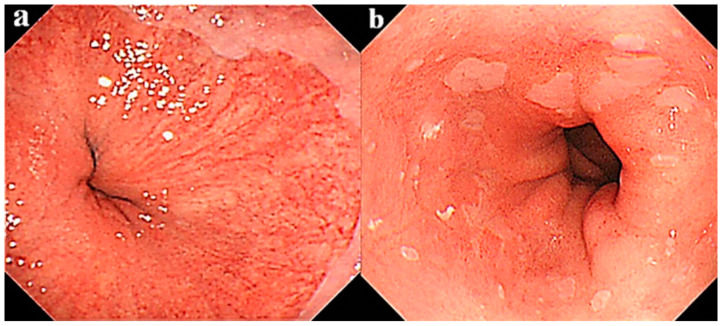
Representative endoscopic findings of patients with segment Barrett’s esophagus (SSBE). Shown are cases with (**a**) visible and (**b**) invisible palisade vessels.

PVs can be endoscopically recognized in the lower esophagus as shown in Figure 1, and the distal end of PVs has been reported to correspond to the esophago-gastric junction [61,68]. PVs are originally located at 2–3 cm range in the lower end of the esophagus in either cases with and without BE. However, PVs become invisible when an intense inflammation is induced by acid and/or bile reflux. Resultantly, PVs were endoscopically detected in 60% of SSBE and only 25% of LSBE patients, respectively [69]. In many LSBE patients, PVs are believed to be endoscopically invisible because of intense inflammation of Barrett’s mucosa. What is the clinical significance of invisible PVs? In our previous study on the relationship between early stage of EAC sized less than 1 cm in diameter and PVs, we have encountered 9 out of 13 (69.2%) patients with superficial EAC who did not showed PVs in whole background mucosa, suggesting that PV disappearance indicates possible malignant potential of BE though an accelerated inflammation [70]. In another study performed thereafter, we compared inflammation grade, expression of COX-2 protein, and cellular proliferation between BE cases with visible and invisible PVs [69]. Two hundred twenty-eight consecutive patients with BE with a length greater than 1 cm were enrolled, and divided into the PVs-visible (n = 154) and PVs-invisible (n = 74, 32.4%) groups. PVs were generally visible in SSBE cases but invisible in most of LSBE ones. In this study, PVs-visible was defined when PVs were possible to be observed in a whole area of BE under an enough extensive condition of the lower esophagus through the deep inspiration to guarantee the inter-observer diagnostic concordance [67,71]. The grade of inflammation was classified according to the updated Sydney system [72] and expression of COX-2 protein was immunohistochemically investigated using a method previously reported [63]. The inflammatory grade was 2.17 ± 0.98 in the PVs-visible group and 3.35 ± 0.69 in the PVs-invisible group (*p* < 0.05), while rates of COX-2 protein expression were 39.0% and 63.5% (*p* < 0.001), respectively. Our analysis showed that inflammatory grade, COX-2 protein expression rate, and cellular proliferation in the PVs-invisible group were significantly greater as compared to the PVs-visible group. Thus, endoscopic findings indicating invisible PVs may reveal the potential of neoplastic progression in patients with BE, and the surveillance biopsy is possibly omitted when PVs are found in whole area of BE.

### 4.2. Assessment by Mucosal Pattern

**Figure 2:**
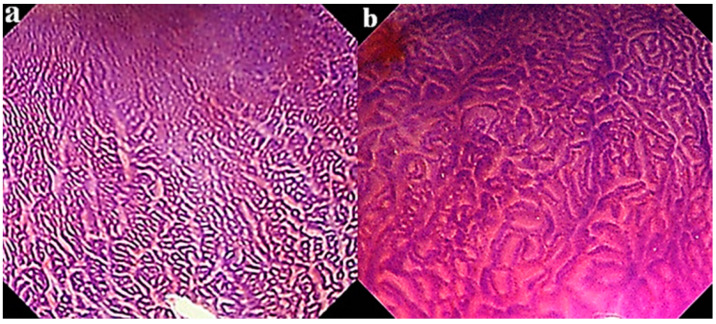
Crystal violet chromoendoscopic views of Barrett’ s esophagus with (**a**) close type and (**b**) open type mucosal pattern.

To investigate the relationship of mucosal pattern with malignant potential, mucosal patterns revealed by crystal violet (CV) chromoendoscopy were divided into closed and open type, as shown in Figure 2 [73,74,75]. A closed type shows a small round pattern, and open type shows a non-round indicating a linear, tubular, villous or irregular one. The COX-2 protein expression rate was 31.9% in the closed type and 67.6% in the open type (*p* < 0.001). Thus, a high rate of COX-2 protein expression may be related to a high inflammatory score with a high cellular proliferative index in BE patients with the open type as compared to those with the closed type of mucosal pattern [76]. Indeed, most of all Barrett’s dysplastic lesions are found in BE patients whose background mucosa showed an open type mucosal pattern, as noted in our previous report [75]. These results consequently may indicate that BE cases with the open type of mucosal pattern possess a malignant potential related to neoplastic progression. However, to clarify whether these markers can be used in clinical settings for determination of malignant potential, a prospective study will be necessary, since these findings were obtained by the retrospective studies.

Mucosal pattern classifications based on CV chromoendoscopy are considered to be similar to the newly developed magnifying NBI endoscopic classification presented by the Japan Esophageal Society for BE (JES-BE) to diagnose dysplastic Barrett’s lesion easier [77]. This JES-BE classification also has divided non-dysplastic BE into pit or non-pit type by the character of mucosal pattern. A pit type mucosal pattern is marked by a circular pattern, and a “non-pit” is marked by non-circular: tubular, linear, or ridged/villous patterns. In that, a non-pit type mucosal pattern is comparable to open type shown by CV chromoendoscopy, which suggests that it may also be possible to detect the malignant potential of BE by use of mucosal pattern detected by NBI endoscopy based on the theory of the expression of COX-2 protein pathway. In a future study, clarification of the JES-BE classification will be needed to determine its effectiveness for detection of the malignant potential of BE.

### 4.3. Assessment of Micro-Vascular Pattern

**Figure 3:**
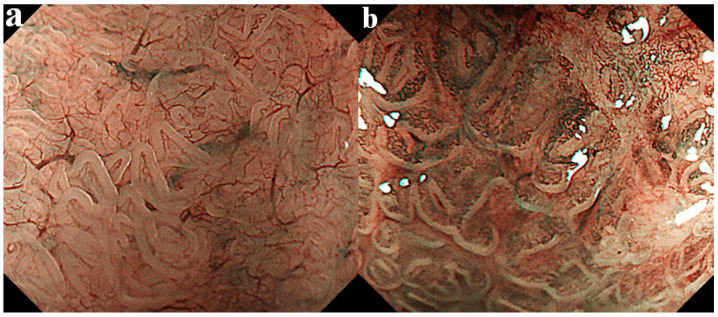
Micro-vascular pattern was divided into the following two categories: type I, uniform branched or vine-like pattern that is able to be traced smoothly (**a**), and type II, coiled or spiral pattern with a nonuniform shape that cannot be traced sufficiently and with increased vascularity (**b**).

Some investigators have reported that oxidative stress concomitant with COX-2 protein expression provokes angiogenesis in the stromal portion of Barrett’s mucosa and subsequently plays an important role in malignant transformation of BE [78,79,80]. We examined micro-vascular density in the stromal portion of Barrett’s mucosa using NBI endoscopy along with image-analyzing software, and based on those findings advocated a new classification based on the micro-vascular pattern consisting of type I and II as shown in Figure 3 [81]. Type I shows uniform branched or vine-like patterns that is able to be traced smoothly, and type II does coiled or spiral patterns with a nonuniform shape that cannot be traced sufficiently. Type II showed a higher micro-vascular density than type I and frequently included the open type of mucosal pattern shown in the CV chromoendoscopy classification. Consequently, the micro-vascular pattern of type II was speculated to be a possible predictor for potential of neoplastic progression, since a higher expression rate of COX-2 protein and accelerated cellular proliferation were proven. Therefore, vascular pattern and/or vessel density revealed by NBI endoscopy may also be useful for predicting the malignant potential in BE cases.

## 5. Endoscopic Findings Showing Possible Predictive Biomarker Information Regarding Initiation and Progression of EAC

### 5.1. Molecular Biomarker Associated with Pathogenesis of EAC Progression

#### 5.1.1. Genetic Instability

Genetic instability, which plays important roles in the initiation and progression of malignant tumors, is closely associated with loss of heterozygosity (LOH), abnormal functions in tumor suppressor genes, changes in DNA methylation, chromosomal aberrations, and dysregulation in cell cycle and signaling [82]. LOH is a common event in patients with allelic imbalance and one of the mechanisms causing inactivation of tumor suppressor genes including p53 that occurs in association with cancer development. A progressive accumulation of LOH in esophageal lesions during the process of malignant transformation has been shown, with endoscopic brush cytology demonstrated to be effective for detection of LOH [83]. Another showed aberrant expression of p53 protein as a representative biomarker for prediction of neoplastic progression in BE [84,85,86,87,88]. Moreover, the *TP53* mutation was found in 46% of patients with EAC progression [25], and reported to be associated with EAC progression with an OR of 3.18 (95% CI 1.68–6.03) [24] and an HR of 6.5 (95% CI 2.5–17.1) [89].

Methylated genes are also considered to have important roles in Barrett’s carcinogenesis [90,91,92]. The expression of p16 methylation, a representative methylated predictor, was reported to contribute to an early event in neoplastic progression, along with expressions of cyclin A and p53 [93]. Hypermethylation of *p16*, *RUNX3*, and *HPP1* genes was found in cases with dysplastic Barrett’s lesions, which possibly occurred at an early stage in BE-associated neoplastic progression, and those three genes was also shown to predict progression risk [91]. Moreover, Ross-Innes CS et al. reported that risk stratification using two methylation markers (*MYOD1*, *RUNX3*) along with three protein biomarkers (p53, c-Myc, Aurora kinase A) was useful to avoid overdiagnosis and overtreatment in routine clinical practice for patients with BE [87].

DNA aneuploidy is considered to be a major chromosomal aberration and one of the important predictors of Barrett’s carcinogenesis [82,87,94]. Fléjou JF noted in a review article that both DNA aneuploidy and polyploidy were strongly related to the presence of EAC [84]. Hadjinicolaou AV et al. also reported that DNA aneuploidy was a stronger predictor of EAC progression than p53 expression [95]. A combination analysis of the three factors: p16 loss, MYC gain, and DNA aneusomy, identified BE cases with a high risk of neoplastic progression, with an HR increased by 8.7-fold (95% CI 2.6–29.8), as compared to those with a low risk of progression [96].

Overexpression of cyclin A is another possible predictor. Moreover, van Olphen SH et al. reported that positive cyclin A expression was associated with increased risk of neoplastic progression, with an RR of 2.4 (95% CI 1.7–3.4) [97], and di Pietro M et al. found that combined expression of cyclin A and p53 was an early event in neoplastic progression [93]. Additionally, *Cyclin A1* DNA methylation was demonstrated to be an efficient method for screening populations at-risk for BE, with high sensitivity and specificity [90].

#### 5.1.2. Growth Factors

Various growth factors are also related to neoplastic progression in BE. A high expression of vascular endothelial growth factor A (VEGFA) was reported to occur in association with neovascularization in BE patients and subsequently induced neoplastic changes [98,99,100]. Inflammation-related biomarkers as well have been investigated. In a study of bile acid-induced inflammation pathways, activation of nuclear factor kappa-light-chain-enhancer of activated B cells (NF-κB) and subsequent DNA damage were indicated based on findings of deoxycholic acid exposure [101]. The relationship of inherited genetic variations with risk of neoplastic progression was also analyzed in an investigation of five inflammation-related pathways (COX, cytokine signaling, oxidative stress, human leukocyte antigen, NF-κB), and the results demonstrated the COX pathway as providing a significant signal regarding BE risk. Moreover, microsomal glutathione S-transferase 1 (MGST1) expression, with elevation of some single nucleotide polymorphisms (SNPs) related to EAC risk in the oxidative stress pathway was also shown [102]. Regenerating gene (*REG*), an epithelial growth factor, has been reported to be involved in chronic inflammation-related carcinogenesis in the stomach and other organs [103,104]. In Barrett’s esophagus, expression of Reg Iα protein was found to induce squamous re-epithelialization of columnar epithelium, suggestive of a suppressive action towards neoplastic progression [105].

#### 5.1.3. Other Markers

A recent meta-analysis of all available genome-wide association studies regarding the relationship between BE and EAC showed a specific association of EAC with the locus near HTR3C and ABCC5, interesting EAC candidate genes that might serve as novel genetic markers for prediction of transition from BE to EAC [106]. In an investigation of aspergillus oryzae lectin (AOL) as a new biomarker, it was demonstrated that not only abnormal DNA ploidy but also AOL could be used to identify BE patients who were most likely to develop dysplastic Barrett’s lesions [92,94]. Janmaat VT et al. also reported that AOL was a useful biomarker to predict neoplastic progression, with an OR of 3.04 (95% CI 2.05–4.49) [24].

### 5.2. Possible Endoscopic Identification of Molecular Biomarker in Association with EAC Progression

There are several biomarkers related to BE carcinogenesis that may be useful for prediction of neoplastic progression in affected patients. Many biomarkers are confirmed by biopsy specimen or brush cytology taken from the target area of BE. However, recent studies have reported some biomarkers that can be detected by use of a unique endoscopic procedure without tissue sampling. For instance, in the current clinical practice, molecular confocal laser endomicroscopy (CLEM), fluorescence imaging with the labeled lectin wheat germ agglutinin (WGA) or near-infrared imaging endoscopy can detect some biomarkers directly. On the other hand, NBI or autofluorescence imaging (AFI) endoscopy are also available to prove the presence of biomarkers as more popular devices, albeit they are indirectly detecting methods. In the area of a tubular or villous mucosal pattern observed by magnifying NBI endoscopy, mRNA expression of MUC2 and/or CDX2 (markers of intestinal differentiation) detected by brushing cytology were the definite evidence of SIM [107]. Moreover, Uno G et al. reported that NBI endoscopy possibly indicated not only COX-2, but also CDX2 and CD34 (angiogenesis-related gene) protein expressions [81]. Thus, NBI endoscopy may reveal biomarkers related to the presence of SIM and stromal angiogenesis.

Molecular imaging endoscopy (MIE) methods, including autofluorescence endoscopy, optical coherence tomography, endocytoscopy, and confocal endomicroscopy, have been shown as novel technologies for detection of biological changes specific to Barrett’s carcinogenesis [82,107]. In these methods, autofluorescence imaging (AFI) endoscopy has been considered to be a representative method for detecting molecular changes associated with EAC progression. Indeed, a feasibility study demonstrated that it was possible to evaluate the status of p53 expression using AFI endoscopic findings [93]. Furthermore, Fels Elliott DR et al. reported that a small panel of biomarkers (low-grade dysplasia, abnormal DNA ploidy, AOL) was capable of identifying BE patients at high risk for developing dysplastic lesions, and concluded that molecular imaging using fluorescently labeled peptides or lectins showed a promise for use as molecular probes in endoscopy to identify dysplastic Barrett’s lesions [85]. Di Pietro M et al. also reported that a three-biomarker panel, composed of aneuploidy, p53 immunohistochemistry, and cyclin A, provided accurate and objective diagnosis of dysplasia in BE patients with a small number of targeted biopsies conducted in an AFI-positive area [108]. However, AFI endoscopy findings demonstrated a high false positive rate as well as low sensitivity, and those disadvantages were subsequently found to cause some difficulties in clinical conditions [82,109]. Furthermore, another report noted that none of the investigated biomarkers showed correlations with AFI findings [93]. On the other hand, fluorescently labeled peptides have been recently developed as molecular probes targeting EAC, while fluorescence imaging with the labeled lectin wheat germ agglutinin (WGA) showed specific binding of WGA to human tissue and consequently successful endoscopic visualization of neoplastic lesions in BE cases [110,111,112].

Confocal laser endomicroscopy (CLEM) has also been applied for endoscopic diagnosis of gastrointestinal neoplastic lesions including Barrett’s dysplastic lesions [113]. Recently, a stratified diagnostic strategy for diagnosis of precancerous and cancerous lesions by CLEM was confirmed to have a high level of accuracy [114], while the additional effect of probe-based CLEM was also proven for detection of dysplastic lesions in BE as compared to magnifying NBI endoscopy alone [115]. Thus, molecular CLEM is considered to be a novel method for diagnosis of dysplastic Barrett’s lesions that allows for visualization of cellular processes in real-time by combinations of a variety of either molecular probes or peptides with fluorescent items. Moreover, near-infrared imaging endoscopy with fluorescence lectin was reported to be capable of differentiating neoplastic from non-dysplastic BE lesions [116]. Nagengast WB et al. also reported that near-infrared imaging endoscopy with use of topical and systemic tracers of VEGFA were useful for diagnosis of neoplastic lesions in BE cases, as a higher detection rate was demonstrated as compared to not only WLE but also NBI endoscopy [117].

Some important biomarkers can only be assessed by use of special endoscopic modalities, though those are complicated and still difficult to apply in usual clinical situations. Moreover, technical and diagnostic variances have been found, even among expert endoscopists. Therefore, further development of endoscopic devices including suitable probes is anticipated.

## 6. What Is an Efficient Method for Surveillance of BE?

Patients with EAC have been gradually increasing in Asian countries, and those with BE are known to share similar risk factors for neoplastic progression as demonstrated in Western populations [118,119]. In Japan, EAC has not been well known in clinical practice, different from esophageal squamous cell carcinoma, though EAC cases have been steadily increasing in association with increases of erosive esophagitis and BE [120]. According to the annual report of the Japanese Association for Thoracic Surgery, the ratio of EAC to squamous cell carcinoma cases had increased to 9.4:100 in 2017 [121]. Moreover, a cohort study conducted by the Japan Gastroenterological Endoscopy Society showed that the annual rate of incidence of EAC arising from Barrett’s esophagus greater than 3 cm in length was 1.2%, though this study was a small number and a short period observation [122]. Therefore, Japanese endoscopists have recently been encouraged to pay adequate attention to accurate and effective management of patients with BE.

A multicenter study conducted in Japan (Japan EAC study) reported that 247 (79%) of 311 superficial Barrett’s carcinoma cases were derived from SSBE, and the total rate of lymph node and/or another organ metastasis was 15.7%, with a 5-year survival rate of 81% [123]. Therefore, some SSBE patients should be followed regularly as same as those with LSBE, albeit all the SSBE cases do not require strict annual surveillance. An efficient survey system is essential for consideration of grading for risk of neoplastic progression.

In Western countries, a random biopsy procedure with 4 quadrants and 1 or 2 cm intervals is recommended for endoscopic surveillance of BE [8,9], though that is time consuming and expensive, and also increases risk associated with the large number of biopsy samples obtained. Given that endoscopy findings can predict the malignant potential of BE, they may provide a more efficient and feasible surveillance method in clinical practice. Based on findings presented in this review, we concluded that a simpler surveillance method to avoid unnecessary biopsy procedures may be possible by use of endoscopic findings with WLE, IEE, and MIE. Sharma P et al. reported that a NBI targeted biopsy can detect more areas with dysplasia as compared to WLE with random biopsied areas [124]. Although a model to determine clinical risk factors of neoplastic progression in patients with BE has already been demonstrated [125], it will be necessary to conduct additional analyses of endoscopic findings to reveal more suitable and novel techniques. Thus, in near future, it may be allowed to propose that a targeting biopsy method combined with IEE and/or MIE plays an important role in surveillance yield of BE cases instead of the Seattle protocol.

## 7. Conclusions

The risk of neoplastic progression in BE appears to be predictable by WLE, IEE, and MIE observations. It is anticipated that a predictive endoscopic scoring system to detect potential neoplastic progression in affected patients will be established in the near future.

## Figures and Tables

**Table 1:** Prediction by WLE findings for neoplastic progression in BE cases.

WLE Findings		Risk Ratio (95% CI)	Study
BE length	per every 1 cm	OR 1.39 (1.34–1.74)	Weston (2004) [30]
		RR 1.11 (1.01–1.20)	Sikkema (2011) [20]
		RR 1.16 (1.03–1.30)	Rugge (2012) [31]
		OR 1.19 (1.09–1.30)	Pohl (2013) [32]
		OR 1.21 (1.12–1.30)	Anaparthy (2013) [33]
		HR 1.16 (1.04–1.30)	Solanky (2019) [34]
	3–8 cm vs. ≥8 cm	OR 2.3 (1.4–3.9) vs. 4.3 (2.5–7.2)	Holmberg (2019) [35]
	LSBE vs. SSBE	OR 2.69 (1.48–4.88)	Pohl (2013) [32]
		HR 7.1 (1.72–29.04)	Coleman (2014) [36]
		No evidence	Bhat (2011) [38]
	SSBE vs. LSBE	HR 0.32 (0.18–0.57)	Hamade (2019) [37]
Barrett’s ulcer		RR 7.60 (2.63–21.9)	Rugge (2012) [31]
		HR 1.72 (1.08–2.76)	Coleman (2014) [36]
Esophagitis		RR 3.5 (1.3–9.5)	Sikkema (2011) [20]
		No evidence	Coleman (2014) [36]
Nodularity		HR 4.98 (1.80–11.7)	Solanky (2019) [34]
Stricture		No evidence	Coleman (2014) [36]
Hiatal hernia	≥6 cm vs. none	OR 17.30 (2.58–115.93)	Weston (2004) [30]
	≥6 cm vs. ≤2 cm	OR 8.55 (1.18–61.56)	
	≥6 cm vs. 3–5 cm	No evidence	
	presence vs. absence	OR 1.2 (1.04–1.39)	Avidan (2002) [42]
		No evidence	Sikkema (2011) [20]
		No evidence	Pohl (2013) [32]
		No evidence	Coleman (2014) [36]
Esophagitis, Ulcer, Nodularity, or Stricture	one marker	HR 6.7 (1.3–35)	Hillman (2003) [41]
	two or more markers	HR 14.1 (2.02–102)	
	Esophagitis + ulcer	HR 8.9 (1.1–75)	
	Nodularity + stricture	HR 17.1 (1.8–162)	

WLE white light endoscopy, BE: Barrett’s esophagus, LSBE: long segment Barrett’s esophagus, SSBE: short segment Barrett’s esophagus, RR: relative risk, OR: odds ratio, HR: hazards ratio, CI: confidence interval.
